# Strengthening Mental Health Though Resilience in Nursing Students: A Protocol for a Comprehensive Scoping Review

**DOI:** 10.3390/nursrep14040248

**Published:** 2024-11-10

**Authors:** Emilia Batista Mourão Tiol, Rauer Ferreira Franco, Amanda Oliva Spaziani, Gabriela Gouvea Silva, Emerson Roberto dos Santos, Vânia Maria Sabadoto Brienze, Alba Regina de Abreu Lima, Sônia Maria Maciel Lopes, Josimerci Ittavo Lamana Faria, Alexandre Lins Werneck, Nádia Antônia Aparecida Poletti, Rafael Guerra de Aquino, Adriana Luiz Sartoreto Mafra, Andreia Mura Peres, Elena Carla Batista Mendes, Thaisa Fernanda Queiroz de Souza, Valéria da Silva Campoi, Luiz Fernando Campoi, Silvia Regina dos Santos Benitez, Patrícia Freire de Vasconcelos, Júlio César André

**Affiliations:** 1 Center for Studies and Development of Health Education, Faculty of Medicine of São José do Rio Preto (CEDES/FAMERP), São José do Rio Preto 15090-000, Brazil; emerson.santos@edu.famerp.br (E.R.d.S.); vania.brienze@hospitaldebase.com.br (V.M.S.B.); alba.lima09@gmail.com (A.R.d.A.L.); sonia.lopes@edu.famerp.br (S.M.M.L.); josimerci.faria@edu.famerp.br (J.I.L.F.); alexandre.werneck@famerp.br (A.L.W.); nadia@famerp.br (N.A.A.P.); julio.andre@edu.famerp.br (J.C.A.); 2 Faculty of Medicine of São José do Rio Preto, São José do Rio Preto 15090-000, Brazil; rauer.franco@edu.famerp.br (R.F.F.); amanda.spaziani@edu.famerp.br (A.O.S.); gabriela.gouvea@edu.famerp.br (G.G.S.); 3 Nursing Department, Santa Fé do Sul University Center (UNIFUNEC), Av. Mangara, 477-Jd. Mangará, Santa Fé do Sul 15775-000, Brazil; rgaquino@funecsantafe.edu.br (R.G.d.A.); alsmafra@funecsantafe.edu.br (A.L.S.M.); amperes@funecsantafe.edu.br (A.M.P.); enfermagem@funecsantafe.edu.br (E.C.B.M.); thaisaqsouza@funec.edu (T.F.Q.d.S.); vscampoi@funecsantafe.edu.br (V.d.S.C.); lfcampoi@funecsantafe.edu.br (L.F.C.); saberviver@santafedosul.sp.gov.br (S.R.d.S.B.); 4 Institute of health sciences, University of Internacional Integration of Afro-Brazilian Lusofonia, Av. da Abolição, 3, Centro, Redenção 62790-000, Brazil; patriciafreire@unilab.edu.br

**Keywords:** nursing students, psychological resilience, mental health, scoping review protocol

## Abstract

Background: Nursing students face unique challenges during their university education, making them vulnerable to mental health problems. Psychological resilience has been identified as a protective factor against these issues. However, previous reviews have identified gaps in the literature on resilience and mental health among nursing students. Objectives: This scoping review aims to identify and map studies on psychological resilience and mental health in undergraduate nursing students, synthesize current evidence on their relationship, identify interventions for enhancing resilience, and highlight gaps in the existing literature. Eligibility criteria: Studies published between January 2019 and April 2024 in English, Portuguese, and Spanish addressing resilience and mental health in undergraduate nursing students will be included. Primary studies, secondary studies, clinical guidelines, and grey literature will be considered. Sources of evidence: Searches will be conducted in multiple databases including EMBASE, ERIC, PubMed, Science Direct, Web of Science, DOAJ, ELSEVIER, EMERALD, and WILEY ONLINE LIBRARY. Grey literature sources will also be searched. Charting methods: Data will be extracted using a standardized form and synthesized narratively. Thematic analysis will be conducted using MAXQDA software ((Verbi GmbH, 24 version, 2023). Quantitative summaries, visual mapping, subgroup analyses, and trend analyses will be performed where appropriate. Results: As this is a protocol, results are not yet available. The review will present a comprehensive map of the current literature on psychological resilience and mental health in nursing students, including identified interventions and research gaps. Conclusions: This scoping review will provide valuable insights to guide curriculum development, support services, and policy-making in nursing education. The findings may support actions to strengthen resilience and prevent mental health problems among future nursing professionals.

## 1. Introduction

The transition to university life can be a challenging experience for students, especially those in healthcare fields such as nursing [1,2]. In addition to academic changes, these students face specific stressors, including uncertainty, excessive workloads, and fear of making mistakes [3]. Consequently, more than half of nursing students are at risk of psychological distress and are vulnerable to mental health problems, such as depression and anxiety [4,5,6].

Recent studies have further emphasized the significance of this issue. A 2023 systematic review by Zhang et al. found that the prevalence of depression among nursing students ranged from 34% to 64% across different countries, highlighting the global nature of this concern [7]. Additionally, a large-scale study conducted in 2024 by Nguyen-Thi et al. reported that nursing students experienced significantly higher levels of stress and anxiety compared to their peers in other healthcare disciplines [8].

In this context, psychological resilience emerges as a crucial protective factor. Resilience is the ability to adapt and recover from adverse situations, reducing the negative effects of stress [9,10]. Nursing students with high resilience tend to have a lower risk of depression and anxiety, being protected from negative academic impacts and psychological distress [5,6,11,12]. A 2024 longitudinal study by Strout et al. demonstrated that nursing students with higher resilience scores at the beginning of their program were 40% less likely to develop symptoms of burnout by their final year [13].

However, previous reviews have identified gaps in the literature on resilience and mental health among nursing students. Alatawi et al. (2022) conducted a scoping review limited to English databases and did not include grey literature [14]. Walsh et al. (2020) identified the importance of developing resilient qualities through appropriate learning opportunities in nursing curricula [15]. More recently, a 2023 systematic review by Han and Yeun called for a more comprehensive mapping of interventions aimed at enhancing resilience in nursing students [16].

Given these facts, it is imperative to conduct further studies to fill these gaps and gain a deeper understanding of the protective role of resilience in nursing students’ mental health. A scoping review approach is particularly appropriate for this topic due to the breadth and complexity of the subject matter, the heterogeneity of study designs in this field, and the need to identify knowledge gaps and future research directions [17]. This scoping review aims to identify and map existing studies on psychological resilience and mental health in undergraduate nursing students to inform future research, practice, and policies in this area.

Specifically, we seek to

1. Synthesize the current evidence on the relationship between psychological resilience and mental health outcomes in nursing students.

2. Identify and categorize interventions designed to enhance resilience and improve mental health among nursing students.

3. Map the geographical distribution of research in this area to identify potential cultural or regional variations.

4. Highlight gaps in the current literature to guide future research priorities.

5. Provide a comprehensive overview to inform the development of evidence-based strategies for promoting resilience and mental well-being in nursing education programs.

By addressing these specific objectives, this scoping review will provide valuable insights to guide curriculum development, support services, and policy-making in nursing education, ultimately contributing to the improved mental health and well-being of future nursing professionals.

## 2. Materials and Methods

This scoping review will be conducted in accordance with the Joanna Briggs Institute (JBI) guidelines for scoping reviews [18] and will follow the steps proposed by Arksey and O’Malley (2005) [19] and Levac et al. (2010) [20]: (1) identifying the research question; (2) identifying relevant studies; (3) study selection; (4) data extraction; (5) collating, summarizing, and reporting the results. This protocol follows the PRISMA-ScR guideline.

### 2.1. Research Question

The research question was formulated using the PCC (Population, Concept, Context) strategy (Table 1): “What has been published in the literature about psychological resilience and mental health among undergraduate nursing students?”.

### 2.2. Eligibility Criteria

We will include studies published between January 2019 and April 2024 in English, Portuguese, and Spanish addressing resilience and mental health in undergraduate nursing students. The following types of studies will be considered:

✓ Primary studies: randomized controlled trials, non-randomized controlled trials, cohort studies, case-control studies, cross-sectional studies, and qualitative studies;
✓ Secondary studies: systematic reviews and meta-analyses;
✓ Clinical guidelines;
✓ Grey literature: conference proceedings, theses, and dissertations.

Studies will be excluded if they

✓ Focus on populations other than undergraduate nursing students;
✓ Do not address psychological resilience or mental health;
✓ Are published in languages other than English, Portuguese, or Spanish;
✓ Are duplicate publications, letters to the editor, editorials, or opinion articles.

### 2.3. Search Strategy

Searches will be conducted in the following databases: EMBASE, ERIC, PubMed, Science Direct, Web of Science, DOAJ, ELSEVIER, EMERALD, and WILEY ONLINE LIBRARY. A search for grey literature will also be conducted [21]. The search strategy will use descriptors in English, Portuguese, and Spanish, combined with Boolean operators (AND, OR, NOT). Table 2 presents an example of the search strategy for PubMed.

### 2.4. Study Selection and Data Extraction

Study selection will be performed in two stages:

1. Title and abstract screening: Two independent reviewers (E.B.M.T. and R.F.F.) will screen titles and abstracts of all retrieved studies against the eligibility criteria.
2. Full-text review: The full texts of potentially eligible studies will be retrieved and independently assessed by two reviewers (A.O.S. and G.G.S.).

Discrepancies at any stage will be resolved through discussion or by a third reviewer if necessary (J.C.A.). The entire selection process will be managed using Covidence software [22]. The selection process will follow the PRISMA-ScR recommendations and will be presented in a flowchart. Data will be extracted using a standardized form, including information on authorship, year, country, objective, study design, population, intervention, comparator, outcomes, results, and conclusions (Table 3).

### 2.5. Quality Appraisal

While a formal risk of bias assessment is not typically conducted in scoping reviews, we will perform a basic quality appraisal of the included studies to provide context for the interpretation of our findings. This appraisal will be conducted at the study level using the Mixed Methods Appraisal Tool (MMAT) developed by Hong et al. (2018) [23].

Two independent reviewers (A.M.P. and E.C.B.M.) will conduct the quality appraisal, with any disagreements resolved through discussion or consultation with a third reviewer (J.C.A.). The results will be presented in a table format, summarizing the methodological strengths and limitations of the included studies.

This quality appraisal is not intended to exclude studies or weigh their findings but rather to provide a comprehensive overview of the current state of the literature on psychological resilience and mental health in nursing students. The information will be used in the data synthesis to contextualize our findings and identify potential areas where the quality of evidence may be lacking, which could inform recommendations for future research.

### 2.6. Data Synthesis and Presentation

Data synthesis will primarily be narrative, complemented by tables and graphs to summarize the findings [24]. We will employ the following methods to handle and summarize the charted data:

1. Thematic analysis: We will conduct a thematic analysis to identify and categorize key themes related to resilience and mental health in nursing students. This process will involve coding the extracted data, identifying patterns, and developing overarching themes.
2. Quantitative summary: Where possible, we will present quantitative summaries of the data, including frequencies and percentages of study characteristics, interventions, and outcomes.
3. Visual mapping: We will use visual representations, such as bubble plots or heat maps, to illustrate the geographical distribution of studies and the relationships between key concepts.
4. Subgroup analysis: We plan to conduct subgroup analyses based on (a) Study design (e.g., experimental vs. observational studies), (b) Geographical region, (c) Type of intervention (for studies examining resilience-enhancing programs), and (d) Year of study in nursing program.
5. Sensitivity analysis: We will perform sensitivity analyses to assess the impact of studies with lower-quality appraisal scores on our findings.
6. Trend analysis: We will examine trends in research focuses, methodologies, and outcomes over time.
7. Gap analysis: We will identify and report on gaps in the existing literature, highlighting areas where further research is needed.
8. Conceptual framework development: Based on the synthesized data, we will attempt to develop a conceptual framework illustrating the relationships between psychological resilience, mental health, and related factors in nursing education.

We will use MAXQDA software for qualitative data analysis [25,26] and SPSS for quantitative analyses. The synthesis will be conducted collaboratively by the review team, with regular meetings to discuss emerging findings and ensure consistency in interpretation.

This comprehensive approach to data synthesis will allow us to provide a nuanced understanding of the current state of knowledge regarding psychological resilience and mental health in nursing students, identify trends and gaps in the literature, and inform future research directions and educational practices.

This review will follow the PRISMA-ScR recommendations to ensure transparency and quality of reporting [27]. The protocol will be registered in the Open Science Framework (OSF) [28].

### 2.7. Timeline

The estimated timeline for this scoping review is as follows:

✓ Protocol development and registration: 2 months;
✓ Literature search and study selection: 3 months;
✓ Data extraction and analysis: 3 months;
✓ Manuscript writing and submission: 2 months;
✓ Total estimated time: 10 months.

### 2.8. Outcomes of Interest

This scoping review will focus on the following main and additional outcomes:

Main Outcomes:
  1. Psychological Resilience Measures: Quantitative or qualitative assessments of nursing students’ ability to adapt positively to adversity or stress.
  2. Mental Health Indicators: Measures of psychological well-being or distress, including but not limited to depression, anxiety, stress, and burnout levels among nursing students.
  3. Interventions for Enhancing Resilience: Any program, strategy, or approach designed to improve psychological resilience in nursing students.

Additional Outcomes:
  4. Academic Performance: Measures of nursing students’ educational achievements, such as grades or clinical competence evaluations.
  5. Attrition Rates: The percentage of nursing students who leave their program before completion.
  6. Coping Strategies: Methods used by nursing students to manage stress and adversity.
  7. Cultural and Contextual Factors: Sociocultural elements that may influence resilience and mental health in nursing students.

These outcomes have been prioritized based on their relevance to our research questions and their potential to inform nursing education practices and policies. The main outcomes directly address our primary objectives, while the additional outcomes provide a broader context for understanding the interplay between resilience, mental health, and nursing education.

This addition will provide clarity on the specific outcomes of the scoping review, enhancing the methodological rigor of the study.

## 3. Discussion

This scoping review will contribute to understanding the relationship between psychological resilience and mental health in nursing students. The results may inform the development of interventions and strategies to promote resilience and mental well-being among these students, with implications for practice, research, and policies in nursing education.

## 4. Conclusions

This scoping review will map the existing literature on psychological resilience and mental health in nursing students, identifying gaps and guiding future research. The findings may support actions to strengthen resilience and prevent mental health problems among future nursing professionals.

## 5. Limitations

This scoping review protocol acknowledges several potential limitations. The language restriction to English, Portuguese, and Spanish may introduce selection bias, potentially omitting relevant research published in other languages. The defined time frame (January 2019 to April 2024) may exclude earlier significant studies, although it aims to focus on the most recent evidence. Additionally, the heterogeneity of included study designs may present challenges in synthesizing and comparing results. As is typical in scoping reviews, a formal quality assessment of included studies will not be conducted, which may limit the ability to evaluate the robustness of the presented evidence.

Furthermore, despite efforts to include grey literature, publication bias remains a risk, with potentially underrepresented negative or null findings. The generalizability of results may be limited due to differences in educational systems and cultural contexts across countries. Lastly, given the dynamic nature of mental health and resilience research, new evidence may emerge during the review process that will not be captured. These limitations will be carefully considered in the interpretation and discussion of the review findings and, where possible, addressed through subgroup or sensitivity analyses.

## Figures and Tables

**Table 1:** PCC question.

Population	Undergraduate nursing students
Concept	Psychological resilience, Mental health
Context	Undergraduate nursing education

Source: Author.

**Table 2:** Preliminary search strategy for PubMed.

Base	Search Strategy	Applied Restrictions	Number of Results
PUBMED	(‘Resilience, Psychological’ OR ‘Psychological Resilience’ OR ‘Resiliency, Psychological’ OR ‘Psychological Resiliency’) AND (‘Mental Health’ OR ‘Health, Mental’ OR ‘Mental Hygiene’ OR ‘Hygiene, Mental’) AND (‘Students, Nursing’ OR ‘Pupil Nurses’ OR ‘Student, Nursing’ NOT ‘Nurses, Pupil’ OR ‘Nurse, Pupil’ OR ‘Pupil Nurse’ OR ‘Nursing Student’ OR ‘Nursing Students’)	- Text availability: Full text - Article type: All types - Publication date: January 2019–April 2024 - Language: English, Portuguese, Spanish - Age: Not restricted - Species: Humans	629 (20 May 2024)

Source: Author.

**Table 3:** Preliminary data extraction form.

Data Extraction Form
General Information 1. Reviewer ID:___________________________________________________________________ 2. Date of data extraction: _________________________________________________________ 3. Study ID: _____________________________________________________________________ 4. Authors: ______________________________________________________________________ 5. Year of publication: ____________________________________________________________ 6. Country: ______________________________________________________________________ 7. Funding source: _______________________________________________________________ Study Characteristics 8. Study design: ( ) Randomized controlled trial ( ) Non-randomized controlled trial ( ) Cohort study ( ) Case-control study ( ) Cross-sectional study ( ) Qualitative study ( ) Mixed-methods ( ) Other: ___________________________________________________ 9. Study setting (specify type of undergraduate nursing education environment): _________________________________________________________________ 10. Sample size: _________________________________________________________________ 11. Participant characteristics: - Age (mean, SD, range): __________________________ - Gender (% male, % female): _____________________________________ - Year of study: ____________________ - Other relevant characteristics: _______________________________ Primary Concepts 12. Psychological resilience measures: ( ) Resilience scale scores ( ) Coping strategies ( ) Adaptability ( ) Other: _________________________________________________________________ 13. Measurement tools used for resilience: _________________________________ 14. Mental health aspects addressed: ( ) Depression ( ) Anxiety ( ) Stress ( ) Burnout ( ) Well-being ( ) Other: _____________________________________________________________ 15. Measurement tools used for mental health: _____________________________ Intervention (if applicable) 16. Type of intervention: __________________________________________________________ 17. Duration of intervention: ______________________________________________________ 18. Frequency of intervention: _____________________________________________________ 19. Intervention provider: _________________________________________________________ 20. Comparison group (if applicable): _______________________________________________ Outcomes 21. Resilience outcomes assessed: ( ) Resilience scale scores ( ) Coping strategies ( ) Adaptability ( ) Other: _________________________________________________________________ 22. Measurement tools used for resilience outcomes: _________________________________ 23. Time points of outcome assessment: _____________________________________________ Results 24. Main findings on the relationship between resilience and mental health: _____________ 25. Statistical analysis used: _______________________________________________________ 26. Effect sizes (if reported): _______________________________________________________ 27. Subgroup analyses (if applicable): _______________________________________________ 28. Adverse events (if reported): ___________________________________________________ Conclusions and Limitations 29. Main conclusions of the study: _________________________________________________ 30. Limitations of the study: _______________________________________________________ 31. Implications for practice, research, or policy: _____________________________________ Additional Notes 32. Other relevant information or comments: ________________________________________

Source: Author.

## Data Availability

The data presented in this study are available in this manuscript. Further details of the data can be requested from the corresponding author with acceptable justification.

## Public Involvement Statement

No public involvement in any aspect of this research.

## Guidelines and Standards Statement

This manuscript was drafted against the Preferred Reporting Items for Systematic Review and Meta-Analysis (PRISMA) extension for protocols (PRISMA-P).

## References

[1] Bolinski F., Boumparis N., Kleiboer A., Cuijpers P., Ebert D., Riper H. (2020). The effect of e-mental health interventions on academic performance in university and college students: A meta-analysis of randomized controlled trials. Internet Interv..

[2] Auttama N., Seangpraw K., Ong-Artborirak P., Tonchoy P. (2021). Factors Associated with Self-Esteem, Resilience, Mental Health, and Psychological Self-Care Among University Students in Northern Thailand. J. Multidiscip. Healthc..

[3] Ching S.S.Y., Cheung K., Hegney D., Rees C.S. (2019). Stressors and coping of nursing students in clinical placement: A qualitative study contextualizing their resilience and burnout. Nurse Educ. Pract..

[4] Serçe O., Ince S.C., Özkul B., Günüşen N.P. (2021). The predictive role of nursing students’ individual characteristics and psychological resilience in psychological distress. Perspect. Psychiatr. Care.

[5] Devi H.M., Purborini N., Chang H.-J. (2021). Mediating effect of resilience on association among stress, depression, and anxiety in Indonesian nursing students. J. Prof. Nurs..

[6] Li Z.-S., Hasson F. (2020). Resilience, stress, and psychological well-being in nursing students: A systematic review. Nurse Educ. Today.

[7] Vo T.N.M., Chiu H.-Y., Chuang Y.-H., Huang H.-C. (2022). Prevalence of Stress and Anxiety Among Nursing Students. Nurse Educ..

[8] Nguyen-Thi T.-T., Le H.M., Chau T.L., Le H.T., Pham T.T., Tran N.T., Ngo Q.P.M., Pham N.H., Nguyen D.T. (2024). Prevalence of stress and related factors among healthcare students: A cross-sectional study in Can Tho City, Vietnam. Ann. Ig..

[9] Nasir S. (2023). Role of Psychologists in Creating Resilience in Victims of Earthquake. J. Prof. Appl. Psychol..

[10] Sirin Gok M., Aydin A., Baga Y., Ciftci B. (2024). The relationship between the psychological resilience and general health levels of earthquake survivor nursing students in Kahramanmaras earthquakes, the disaster of the century. J. Community Psychol..

[11] Mcdermott R.C., Fruh S.M., Williams S., Hauff C., Graves R.J., Melnyk B.M., Hall H.R. (2020). Nursing students’ resilience, depression, well-being, and academic distress: Testing a moderated mediation model. J. Adv. Nurs..

[12] Spurr S., Walker K., Squires V., Redl N. (2021). Examining nursing students’ wellness and resilience: An exploratory study. Nurse Educ. Pract..

[13] Strout K., Schwartz-Mette R., McNamara J., Parsons K., Walsh D., Bonnet J., O’Brien L.M., Robinson K., Sibley S., Smith A. (2023). Wellness in Nursing Education to Promote Resilience and Reduce Burnout: Protocol for a Holistic Multidimensional Wellness Intervention and Longitudinal Research Study Design in Nursing Education. JMIR Res. Protoc..

[14] Alatawi A.O., Morsy N.M., Sharif L.S. (2021). Relation between Resilience and Stress as Perceived by Nursing Students: A Scoping Review. Evid.-Based Nurs. Res..

[15] Walsh P., Owen P.A., Mustafa N., Beech R. (2020). Learning and teaching approaches promoting resilience in student nurses: An integrated review of the literature. Nurse Educ. Pract..

[16] Han S.-J., Yeun Y.-R. (2023). Psychological Intervention to Promote Resilience in Nurses: A Systematic Review and Meta-Analysis. Healthcare.

[17] Peters M.D.J., Godfrey C., Mcinerney P., Munn Z., Tricco A.C., Khalil H., Aromataris E., Lockwood C., Porritt K., Jordan Z. (2020). Scoping Reviews. JBI Manual for Evidence Synthesis.

[18] Peters M.D.J., Marnie C., Tricco A.C., Pollock D., Munn Z., Alexander L., McInerney P., Godfrey C.M., Khalil H. (2020). Updated methodological guidance for the conduct of scoping reviews. JBI Evid. Synth..

[19] Arksey H., O’Malley L. (2005). Scoping studies: Towards a methodological framework. Int. J. Soc. Res. Methodol..

[20] Levac D., Colquhoun H., O’Brien K.K. (2010). Scoping studies: Advancing the methodology. Implement. Sci..

[21] Paez A. (2017). Gray literature: An important resource in systematic reviews. J. Evid.-Based Med..

[22] Babineau J. (2014). Product Review: Covidence (Systematic Review Software). J. Can. Health Libr. Assoc..

[23] Hong Q.N., Fàbregues S., Bartlett G., Boardman F., Cargo M., Dagenais P., Gagnon M.-P., Griffiths F., Nicolau B., O’cathain A. (2018). The Mixed Methods Appraisal Tool (MMAT) version 2018 for information professionals and researchers. Educ. Inf..

[24] Popay J., Roberts H., Sowden A., Petticrew M., Arai L., Rodgers M., Britten N., Roen K., Duffy S. (2006). Guidance on the Conduct of Narrative Synthesis in Systematic Reviews: A Product from the ESRC Methods Programme.

[25] Borenstein M., Hedges L.V., Higgins J.P.T., Rothstein H.R. (2021). Introduction to Meta-Analysis.

[26] Rädiker S., Kuckartz U. (2020). Focused Analysis of Qualitative Interviews with MAXQDA.

[27] Tricco A.C., Lillie E., Zarin W., O’Brien K.K., Colquhoun H., Levac D., Moher D., Peters M.D.J., Horsley T., Weeks L. (2018). PRISMA Extension for Scoping Reviews (PRISMA-ScR): Checklist and Explanation. Ann. Intern. Med..

[28] Foster E.D., Deardorff A. (2017). Open Science Framework (OSF). J. Med. Libr. Assoc..

